# Gender differences in sleep patterns and sleep complaints of elite athletes

**DOI:** 10.5935/1984-0063.20190084

**Published:** 2019

**Authors:** Andressa Silva, Fernanda Veruska Narciso, João Paulo Rosa, Dayane Ferreira Rodrigues, Aline Ângela da Silva Cruz, Sérgio Tufik, Fernanda Viana, Jorge José Bichara, Sebastian Rafael Dias Pereira, Sidney Cavalcante da Silva, Marco Túlio De Mello

**Affiliations:** 1 Universidade Federal de Minas Gerais, Departamento de Esportes - Belo Horizonte - Minas Gerais - Brazil.; 2 Universidade Federal de São Paulo, Departamento de Psicobiologia - São Paulo - São Paulo - Brazil.; 3 Comitê Olímpico do Brasil, Comitê Olímpico do Brasil - Rio de Janeiro - Rio de Janeiro - Brazil.

**Keywords:** Sleep, Athletes, Sports, Sleep Disorders, Gender

## Abstract

**Objective:**

The present study aimed to investigate the gender differences for sleep complaints, patterns and disorders of elite athletes during preparation for the Rio 2016 Olympic Games.

**Methods:**

The study included 146 athletes from the Brazilian Olympic Team (male: n=86; 59%; female: n=60; 41%). The assessment of the Olympic athletes’ sleep took place in 2015, during the preparation period for the Rio Olympic Games. The athletes underwent a single polysomnography (PSG) evaluation. Sleep specialists evaluated the athletes and asked about their sleep complaints during a clinical consultation. In this evaluation week, the athletes did not take part in any training or competitions.

**Results:**

The prevalence of sleep complaints was 53% of the athletes during the medical consultation, the most prevalent being insufficient sleep/waking up tired (32%), followed by snoring (21%) and insomnia (19.2%). In relation to the sleep pattern findings, the men had significantly higher sleep latency and wake after sleep onset than the women (*p*=0.004 and *p*=0.002, respectively). The sleep efficiency and sleep stages revealed that men had a lower percentage of sleep efficiency and slow wave sleep than the women (*p*=0.001 and *p*=0.05, respectively).

**Conclusion:**

Most athletes reported some sleep complaints, with men reporting more sleep complaints than women in the clinical evaluation. The PSG showed that 36% of all athletes had a sleep disorder with a greater reduction in sleep quality in men than in women.

## INTRODUCTION

The prevalence of sleep disturbances have been documented essentially only in men, however, in sleep surveys, women report considerably more sleep problems than men^1^, and there are few studies in relation to gender and sleep in athletes^2^. Sleep is considered an important aspect of the post-exercise recovery process by coaches and athletes, and a critical factor for optimal performance^3^^,^^4^. Sleep quality is an important factor which deserves to be taken into account in evaluations of high-performance athletes^5^^-^7. Poor sleep quality is common self-report among athletes, particularly before competitions, and can have a significant impact on their performance^8^^,^^9^.

Likewise, sleep restriction and sleep deprivation, common conditions among athletes (ex.: jet lag, night game competitions) can impact maximal aerobic demands and muscular strength and power^8^. Even at circadian time of physical performance acrophase (strength, flexibility, alertness and anaerobic power output) that coincides with the core temperature acrophase^10^^,^^11^.

Furthermore, the sleeping patterns of elite athletes and effects of sleep loss remain uncertain^8^ due some physiological differences (long sleepers *vs.* short sleepers)^12^, training characteristics^2^, and prior competition period^13^. In addition, some differences exist in sleep quality, duration, latency, and architecture between men and women^14^. For example, in sleep architecture slow wave sleep is lower in women as compared with men^15^.

Athletes who suffer from unrefreshing sleep do not enjoy the benefits of restorative sleep^16^. A systematic review conducted by Gupta et al.^6^ demonstrated that pooled sleep quality data revealed high levels of sleep complaints in elite athletes, and found that studies broadly identified three main risk factors for sleep disturbance: training^17^, travel^18^ and competition^5^^,^^19^^,^^20^. According to the review, most studies reporting sleep problems in elite athletes are based on questionnaires and actigraphy^6^, with only two studies using polysomnography (PSG) to evaluate sleep quality, sleep pattern and sleep disturbance^21^^,^^22^.

It was observed in the general population that men and women present difference in sleep pattern and sleep disorders^1^^,^^23^. However, the participation of women has been prominent in elite sports, therefore it is necessary more research in this area. Although some studies have already been developed in this area, it is still unclear the differences between the genres of elite athletes.

Taking into account the scarcity of information in the literature regarding the impact of sleep disorders, particularly in relation to gender, and the fact that few studies have been conducted using PSG^24^, the present study proposed to investigate gender differences in sleep complaints, sleep patterns and sleep disturbances through the clinical and polysomnographic evaluation of Brazilian Olympic Team elite athletes during the preparation for the Rio 2016 Olympic Games.

## MATERIAL AND METHODS

### Participants

The sample was of convenience with elite athletes of the Brazilian Olympic Team from individual sports. In addition, the sample size was similar to other studies^19^^,^^25^. We evaluated 146 elite athletes preparation for the Rio 2016 Olympic Game, (modern pentathlon (n=3), artistic gymnastics (n=22), canoeing (n=5), swimming (n=24), field and track (n=31), judo (n=47), beach volleyball (n=10), sailing (n=4) of both sex (men: n=86; 59% and women: n=60; 41%), with a mean age of 24.3±4.6 years, mean body mass 67.75±2.60kg, mean height of 1.73±0.05m, and mean body mass index (BMI) of 22,66±0.65kg/m^2^, in the preparatory cycle for the Rio 2016 Games through clinical and polysomnographic examination. All participants in the study gave their informed consent. This study was approved by the Ethical Committee of the Universidade Federal de São Paulo (UNIFESP) (Protocol number #0294/11).

### Procedure and Evaluations

A team of sleep specialists evaluated the sleep of the athletes of the Brazilian Olympic Team in the preparatory cycle for the Rio 2016 Games, considering any sleep complaints, sleep pattern or sleep disturbance. Each athlete underwent a single PSG in the preparatory cycle for the Rio 2016 Games, which was scheduled during the medical evaluation week, in which no training or competitions took place so as not to interfere with the sleep evaluations. PSG evaluations were conducted from May 2015 to May 2016, in the preparatory cycle for the Rio 2016 Games.

The athletes underwent the clinical evaluation and the PSG exam at the Sleep Institute (Associação Fundo de Incentivo à Pesquisa - AFIP), São Paulo, Brazil. The Sleep Institute is a national and international reference center staffed by a multidisciplinary group, and the physicians responsible for assessing the sleep disorders used the same standardized questionnaire to evaluate the athletes.

### Clinical Evaluation with a Sleep Specialist

A team of sleep specialists conducted the clinical evaluation using the UNIFESP Sleep Questionnaire developed and validated by the Sleep Institute/AFIP to evaluation of sleep disorders, with insomnia, parasomnias and excessive sleepiness, an overall sensitivity of 70% and specificity of 75% compared by medical examination and polysomnography^26^.

During completion of the questionnaire we asked the athlete about any sleep problems such as waking up tired, having insufficient sleep, insomnia, excessive daytime sleepiness, breathing complaints, restless legs, snoring, moving a lot during sleep, nightmares, light sleep, bruxism, talking in the sleep, and somnambulism. We based the definition for identifying the presence of 1 or more sleep complaints on the athlete’s positive response to the questions, independent of the frequency of the complaint^27^.

After the consultation with the doctor, we sent all the athletes for the polysomnography examination. On the day after the PSG examination, the athlete attended a further appointment with a sleep specialist to be evaluated using the result of the PSG, and to receive advice and guidance on sleep and, if they had been diagnosed with some sleep disorder.

### Polysomnography (PSG)

All athletes underwent a single PSG evaluation during the preparatory stage for the Rio 2016 Games. The athletes slept for one night in the sleep laboratory of the Sleep Institute, São Paulo, Brazil. PSG was performed overnight in a quiet, dark environment using an Embla^®^ S7000 system (Embla Systems Inc., Reykjavik, Iceland). We attached all recording sensors to the patient in a non-invasive manner with tape or elastic bands.

We monitored the following physiological variables simultaneously and continuously: a four-channel electroencephalogram (EEG) (C3-A2, C4-A1, O1-A2, O2-A1), two-channel electrooculogram (EOG) (EOG-Left-A2, EOG-right-A1), four-channel surface electromyography (submental region muscle, tibialis anterior muscle, masseter region and seventh intercostal space) and single-channel electrocardiogram (modified lead V1). Airflow detection was conducted via 2 channels through a pair of thermal sensors (single channel) and nasal pressure (single channel) and respiratory effort of the thorax (single channel) and abdomen (single channel) using inductance plethysmography. Snoring (single channel), position (single channel), oxygen saturation (SaO_2_) and a single pulse oximeter were monitored via EMBLA. All PSG recordings were collected, and sleep stages were scored manually according to the original^28^ standard criteria of the American Academy of Sleep Medicine (AASM)^29^.

### Statistical Analysis

We used descriptive statistics for the data analysis, consisting of standard deviation, frequency distribution and a confidence interval (CI) of 95% for the variables derived from the PSG results in relation to gender differences. In addition, we used information about sleep complaints from the athletes’ individual medical reports. We categorized and analyzed this information using relative frequency. We used the independent t-test to compare PSG variables between genders. We used the Chi-Square test () to detect the existence of any association between the nominal variables and between genders. We considered the probability of a type I error (α) of 5%. The effect size (ES) were interpreted using the scale of magnitudes proposed previously.

## RESULTS

### Sleep complaints reported by the elite athletes

The prevalence of sleep complaints reported by the elite athletes of the Brazilian Olympic Team according to the sleep questionnaire was 53% (77 athletes, 47 men and 30 women) (*p*=0.053). In relation to sleep complaints athletes reported a total of 250, with some athletes having more than one sleep complaint. The most frequent complaint was insufficient sleep/waking up tired 32%, followed by snoring 21.6%, insomnia 19.2%, excessive daytime sleepiness 8.8%, night awakening 5.6% and breathing complaints 4.0%. The least common complaints were moving a lot during sleep 3.2%, restless legs 1.6%, talking in the sleep 1.2%, nightmares 1.2%, bruxism 1.2%, and somnambulism 0.4%. Waking up tired/having insufficient sleep was the main complaint for both genders 32% (80 athletes, men: n=47; 58.8% and women: n=33; 41.2%). Men complained more frequently of snoring than women 21.6% (54 athletes, men: n=37; 68.5% and women=17; 31.5%). However, the snoring was the only complaint with significant differences according to gender, more prevalence men, 68.5%, than women, 31.5% (*p*=0.006). The other complaints presented similar distributions between the genders (Table 1).

**Table 1:** Prevalence of sleep complaints reported by elite athletes of the Brazilian Olympic Team when responding to the sleep questionnaire.

Sleep Complaints	Overall n (%)	Men n (%)	Women n (%)	Comparison
**Insufficient sleep/waking up tired**	80 (32%)	47 (58.8%)	33 (41.2%)	χ2=2.450; *df*=1; *p*=0.118
**Snoring**	54 (21.6%)	37 (68.5%)	17 (31.5%)	χ2 7.407; *df*=1; *p*=0.006*
**Insomnia**	48 (19.2%)	26 (54.2%)	22 (45.8%)	χ2=0.333; *df*=1; *p*=0.564
**Excessive daytime sleepniness**	22 (8.8%)	14 (63.6%)	8 (36.4%)	χ2=1.636; *df*=1; *p*=0.201
**Night awakening**	14 (5.6%)	8 (58.7%)	6 (42.1%)	χ2=0.333; *df*=1; *p*=0.564
**Breathing complaints**	10 (4.0%)	5 (50%)	5 (50%)	χ2=0.000; *df*=1; *p*=1.000
**Moving a lot during sleep**	8 (3.2%)	4 (50%)	4 (50%)	χ2=0.000; *df*=1; *p*=1.000
**Restless legs**	4 (1.6%)	2 (50%)	2 (50%)	χ2=0.000; *df*=1; *p*=1.000
**Talking in sleep**	3 (1.2%)	2 (66.7%)	1 (33.3%)	χ2=0.333; *df*=1; *p*=0.564
**Nightmares**	3 (1.2%)	2 (66.7%)	1 (33.3%)	χ2=0.333; *df*=1; *p*=0.564
**Bruxism**	3 (1.2%)	1 (33.3%)	2 (66.7%)	χ2=0.333; *df*=1; *p*=0.564
**Somnambulism**	1 (0.4%)	1 (100%)	-	χ2=0.000; *df*=1; *p*=1.000

* Results that obtained significance of *p*<0.05 between genders.

### Polysomnography Findings in the Elite Athletes

The PSG data are presented in Table 2, and shows that the men had significantly higher sleep latency and wake after sleep onset than the women. The men had a lower sleep efficiency than the women. Regarding sleep disturbances, the apnea/hypopnea index (AHI) and the microarousal index were significantly higher in the men than in the women. However, we observed no significant differences between genders in total sleep time, REM sleep latency, stage N1, stage N2, slow wave sleep N3, percentage of REM sleep and the periodic leg movement index.

**Table 2:** Polysomnography Findings in the Elite Athletes of the Brazilian Olympic Team.

Parameters	Overall (n=146) Mean±SD (IC95%)	Men (n=86)	Women (n=60)	*p*	EF
**Total sleep time (hours)**	5:31± 0:49 (5:23 to 5:39)	5:26±0:54 (5:14 to 5:38)	5:38±0:39 (5:28 to 5:49)	0.121	0.26
**Sleep latency (min)**	31.36±34.87 (25.66 to 37.07)	37.74±39.83 (29.20 to 46.28)	22.23±23.63 (16.12 to 28.33)	0.004*	0.49
**REM sleep latency (min)**	86.55±39.84 (80.03 to 93.06)	86.98±40.31 (78.34 to 95.62)	85.93±39.48 (75.72 to 96.13)	0.875	0.03
**Wake after sleep onset (min)**	23.25±20.87 (19.83 to 26.66)	27.31±24.34 (22.10 to 32.53)	17.42±12.57 (14.17 to 20.67)	0.002*	0.54
**Sleep efficiency (%)**	86.11±9.85 (84.50 to 87.72)	83.53±10.96 (81.18 to 85.88)	89.80±6.47 (88.13 to 91.48)	0.001*	0.72
**Stage N1 of NREM sleep (%)**	6.69±3.86 (6.06 to 7.32)	7.18±4.0 (6.32 to 8.04)	5.99±3.57 (5.07 to 6.92)	0.067	0.31
**Stage N2 of NREM sleep (%)**	48.01±8.37 (46.64 to 49.38)	47.94±9.08 (46.00 to 49.89)	48.11±7.31 (46.22 to 50.00)	0.907	0.02
**Slow wave sleep N3 (%)**	24.41±7.29 (23.21 to 25.60)	23.43±7.01 (21.93 to 24.93)	25.81±7.52 (23.86 to 27.75)	0.050	0.33
**REM sleep (%)**	20.62±5.29 (19.75 to 21.48)	20.99±5.28 (19.85 to 22.12)	20.09±5.30 (18.71 to 21.46)	0.312	0.17
**Arousal (frequency)**	45.77±25.47 (41.61 to 49.94)	46.12±27.89 (40.14 to 52.10)	45.28±21.75 (39.66 to 50.90)	0.847	0.03
**Arousal index (frequency/hour)**	3.11±4.90 (2.31 to 3.92)	4.05±6.10 (7.47 to 9.70)	1.78±1.57 (7.03 to 8.97)	0.001*	0.59
**AHI (frequency/hour)**	1.70±4.36 (0.99 to 2.42)	2.58±5.51 (1.40 to 3.76)	0.45±0.54 (0.31 to 0.59)	0.001*	0.70
**PLMI (frequency/hour)**	1.53±6.65 (0.45 to 2.62)	1.22±4.73 (0.20 to 2.23)	1.98±8.71 (-0.27 to 4.23)	0.497	0.11

Data are reported as means ± SD (95% CI) polysomnographic recordings.

* Results that obtained significance of *p*<0.05 between genders. REM = Rapid eye movement; PLMI = Periodic leg movement index; AHI = Apnea-hypopnea index; EF = Effect size.

### Prevalence of sleep disturbances detected by polysomnography and clinical evaluation

In relation to sleep disorders detected by polysomnography and clinical evaluation, no significant differences (*p*>0.05) were found between genders. According to the polysomnographic records and clinical evaluation, 53 (36%) athletes had some sleep disturbance, with more prevalence in men, 35 (68%), than women, 18 (32%) (*p*=0.017). Men had a higher prevalence of insomnia and sleep apnea than women, however, women seem to be more affected by bruxism than men (Table 3).

**Table 3:** Prevalence of sleep disturbance detected by polysomnography (AHI, PLM, bruxism) and clinical evaluation (insomnia).

	Overall n (%)	Men n (%)	Women n (%)	Comparison
**Insomnia**	28 (19%)	18 (64%)	10 (36%)	χ2=2.286; *df*=1; *p*=0.131
**Bruxism**	10 (7%)	4 (40%)	6 (60%)	χ2=0.400; *df*=1; *p*=0.527
**AHI >5 to <15 events/h**	6 (4%)	6 (100%)	0 (0%)	-
**AHI ≥15 to <30 events/h**	3 (2%)	3 (100%)	0 (0%)	-
**AHI ≥30 events/h**	1 (1%)	1 (100%)	0 (0%)	-
**PLM >5 to <25 events/h**	5 (3%)	3 (60%)	2 (40%)	χ2=0.200; *df* =1; *p*=0.655

AHI=Apnea-hypopnea index; PLM=Periodic leg movement.

## DISCUSSION

We examined the gender differences for sleep complaints, sleep patterns and sleep disturbance in the elite athletes of the Brazilian Olympic Team, and observed that the majority of the athletes presented some sleep complaint (53%), being more prevalent in the male (61%) athletes than in the female athletes (39%), however, we observed no significant differences.

The most prevalent sleep complaints being: waking up tired/insufficient sleep, followed by snoring, insomnia and excessive daytime sleepiness. These findings of sleep complaints in the male athletes were confirmed by the PSG examination, which showed that they appeared to be more affected in relation to their sleep pattern, as they presented increased sleep latency, decreased sleep efficiency and slow-wave sleep, and increased arousal and apnea hypopnea indexes compared to the female athletes. The PSG assessment and clinical evaluation showed that 36% of the athletes presented some sleep disturbance, with insomnia being the most common.

In contrast to our data, in general population women reported more sleep problems than men^6^. The differences about sleep between men and women is recognized by the influence of sex hormones on the circadian rhythm^30^. In spite of men and women have similar sleep needs, the risk and type of sleep disorders may vary between the sexes^31^. While women tend to have central sleep disorders (affecting the ability to fall asleep at a reasonable time and stay asleep), men are more tendency to obstructive sleep disorders (sleep apnea)^31^. The evidence of the sleeping patterns of elite athletes during the training and competition cycle still remain unclear and more research considering the variability of sleep among elite athletes are needed, related not only to the gender differences, but others parameters such as for example athletes from individual or team sports to elucidate processes linked to the recovery and performance of elite athletes.

The fact that we found sleep complaints in 53% of the athletes is in line with the findings in the literature, which show a similar percentage of sleep complaints such as snoring, insomnia, insufficient sleep and bruxism in the general population. A study of the population of São Paulo, the largest city in Brazil^1^^,^^23^, showed a similar result and also demonstrated that sleep complaints were more prevalent in men than in women. Snoring was the most prevalent complaint in the study, with an increase of 20% over the previous 2 decades^23^.

This complaint is also present in athletes^19^. Possible factors can predicting males report snoring more than females, such as age, large neck size, high BMI, large body mass, anatomic features^19^^,^^32^ . The study also found that 63% of the population sampled reported at least 1 sleep related complaint^33^. In addition, a study by Tuomilehto et al.^34^ reported that sleep problems are common in professional athletes.

Sleep disorders are therefore common, with approximately one-third of adults in the general population complaining of insomnia, but sleep can also be disturbed over short periods as a result of stress and particular stressful events^35^. Swinbourne et al.^19^ investigated the prevalence of poor sleep quality, sleepiness and obstructive sleep apnea risk factors in athletes and the findings suggest that this cohort of team sport athletes suffer a preponderance of poor sleep quality, with associated high levels of daytime sleepiness.

In the present study, when we considered the PSG records and clinical evaluation together, 36% of all athletes had a diagnosed sleep disorder, which is in fact consistent with the literature. Our research group conducted a study to evaluate sleep quality using the Pittsburgh questionnaire with track and field athletes before the 2008 Beijing Paralympic Games, and observed that most of them had poor sleep quality^1^.

Regarding the findings of the present study, we observed a total sleep time were 5.3 hours in the elite athletes, for ideal is around 7-8 hours^2^^,^^17^^,^^19^.This pattern of degraded sleep quality is clearly illustrated by the composite measure of sleep efficiency and total sleep time derived from actigraphy studies reported in the study by Gupta et al., who also found that elite athletes had fewer hours sleep compared to non-athletes^2^^,^^6^^,^^36^. Six Australian swimmers selected to participate in the 2008 Beijing Olympics exhibited significantly different wake-sleep behaviors on training days and resting days. On the nights before training days, athletes slept an average of 5.4 hours. On the nights before rest days, the average total sleep time was 7.1 hours^17^. Recently published studies^2^^,^^17^^,^^19^^,^^25^^,^^37^, found the mean total sleep duration of athletes to be around 7 hours; these studies highlighted the importance of sleep for an athlete’s performance. In general, athletes had a lower total sleep duration than non-athletes^2^.

These findings are important given that consecutive nights of reduced total sleep duration can lead to deficits in neurobehavioral performance, increased daytime sleepiness and fatigue^38^^-^40 and reduced testosterone levels^41^. In contrast, regular sleep, and sleeping 7-8 hours per night results in quicker reaction times and lower levels of daytime sleepiness and fatigue^42^. It may be possible that seven hours of sleep is not enough time for these athletes to recover from their high training loads^43^, with competitions and training routines requiring a greater need for recovery by the athletes^2^^,^^17^. Based on these findings the athletes should receive education on how to improve sleep wake schedules, extend total sleep time and improve sleep quality.

In the context of elite sport, reported incidences of insomnia involved factors which justify specific attention to sleep quality (in addition to sleep structure and patterns) when reviewing sleep and sport interactions^6^. This can also be observed in our study, as we found increased sleep latency, decreased sleep efficiency and the slow wave sleep (Stage 3) in the men. These are novel findings and suggest that male athletes may require more assistance in relation to sleep than female athletes. In other studies^44^^-^46, the impact of exercise on sleep has most frequently been expressed in terms of post-exercise changes in sleep-stage organization, with increases in levels of exercise leading to greater duration and intensity of stage 3 sleep. It has long been recognized, however, that polysomnographic macrostructure (as reflected in standard sleep stages) poorly discriminates between those who report good quality sleep and those who report symptoms of insomnia^47^.

In our study, considering the PSG and clinical evaluation results together, the most prevalent sleep disturbance in the athletes was insomnia (19%) and was more prevalent in the men than women. The findings of the present study corroborate the systematic review carried out by Gupta et al.^6^ who reported that the current literature consistently reports that elite athletes generally show a high overall prevalence of insomnia symptoms, characterized by longer sleep latencies, greater sleep fragmentation, non-restorative sleep, and excessive daytime fatigue.

Sleep disorders differences between men and women are observed in the general population^1^^,^^23^, however in elite athletes, there are controversies. In the study by Schaal et al. the highest rate of sleep disorders are in women, having a greater difficulty of initiating and maintaining sleep^48^, and a study by Leeder et al.^2^ demonstrated that male athletes had a greater total sleep time when compared to female athletes. However, the study by Juliff et al.^13^ there was no difference between both sex, although athletes reported a high rate of pre-competition sleep disorders.

Periods of competition and training appear to perpetuate sleep disruptions; however, sleep disruptions reported in response to such sleep challenges exhibit high variability, as well as highlighting two mechanisms in particular associated with sport related insomnia symptoms, and therefore potential targets for intervention: pre-sleep cognitive/psycho-physiological arousal, and sleep restriction. There is increasing evidence that sleep interventions can improve the quality and extend the duration of athletes’ sleep^49^.

Therefore, it is important to determine for each athlete their required total sleep time, the presence of any sleep disturbances/fragmentation, and their particular athlete’s preferred circadian rhythm, as well as there are factors that affect post-exercise recovery and performance^16^, as the secretion of human hormones. Studies show that when an athlete does not sleep properly, the production of this hormone decreases^50^.

As poor sleep quality and sleep loss interferes on the performance and reduces muscle recovery^8^^,^^51^^-^52, consequently, these factor may increase musculoskeletal injuries^16^^,^^53^. All these changes in the sleep patterns of elite athletes can therefore negatively affect performance and increase injuries.

The limitations of the present study include the fact that athletes performed only one PSG, we performed no follow-up of athletes to find out if there was an improvement in their sleep following the intervention, and that we did not analyses the results according to the athletes sport. We conclude that most athletes reported some sleep complaints, with men reporting more sleep complaints than women in the clinical evaluation, men were more likely to snore and have insomnia than women. In addition, PSG showed that 36% of all athletes had a sleep disorder and sleep quality was worse in men than in women, in particular in relation to sleep latency, arousal index, apnea-hypopnea index, sleep efficiency and slow wave sleep. Therefore, to achieve optimal performance, particular attention should be paid to the sleep quality of athletes, especially when preparing for important competitions, as was done in the preparation of the Brazilian team for the 2016 Olympic Games.
